# Nationwide incidence of congenital and infantile cataract requiring surgery in Korea

**DOI:** 10.1038/s41598-024-53339-y

**Published:** 2024-03-04

**Authors:** Dong Geun Kim, Da Yun Lee, Se Joon Woo, Kyu Hyung Park, Sang Jun Park

**Affiliations:** 1 Department of Ophthalmology, Seoul National University College of Medicine, Seoul National University Bundang Hospital, Seongnam, South Korea; 2 Department of Ophthalmology, Inje University College of Medicine, Inje University Busan Paik Hospital, Busan, South Korea; 3 Department of Ophthalmology, Seoul National University College of Medicine, Seoul National University Hospital, Seoul, South Korea

**Keywords:** Eye diseases, Public health

## Abstract

Congenital and infantile (CI) cataract is one of the most important and preventable cause of blindness in children, but the incidence has not been studied in Korea. We collected data from the national claims database of the National Health Insurance Service of Korea from 2002 through 2019. We identified children who underwent cataract surgery within the age of 5 years, and cumulative incidence rates were calculated for each of the three age criteria. 989 patients out of 4,221,459 births underwent surgery with CI cataract during the period. The cumulative incidence rates per 10,000 births were 1.60 (0–1 years), 2.38 (0–3 years), and 2.95 (0–5 years), respectively. The incidence peaked in the 2007 birth cohort, which coincides with the start of the national screening program for infants/children. Primary intraocular lens implantation was performed in 439 patients (44%). Strabismus and glaucoma requiring surgery occurred in 291 patients (29.4%) and 32 patients (3.2%), respectively, within 8 years after cataract surgery. The incidence rates of CI cataract in Korea appear to be comparable to previous studies in other regions. The early screening program for infants may reduce delayed diagnosis and increase the proportion of patients undergoing surgery at a critical time for visual development.

## Introduction

Cataract refers to an opacity of the crystalline lens, which can blur or block the image reaching the retina depending on the severity. Although cataract in early childhood is quite rare but should not be overlooked, because blocking or blurring of visual stimuli during the critical period of visual development can cause irreversible deprivation amblyopia and permanent severe visual impairment^1^. So childhood cataract is one of the most important and preventable cause of blindness in children, and accounts for 5–20% of pediatric blindness worldwide^2^.

Previous reports suggested that approximately 200,000 children are blind due to cataracts worldwide and 20,000 to 40,000 children are born with cataract each year, which varied widely depending on the region throughout the world^3–5^. The results of these literatures suggest that a sufficiently large population and accurate patient definition are necessary to produce an accurate incidence rate due to the low incidence of childhood cataract. However, the incidence or prevalence of congenital and infantile cataracts, which account for most of childhood cataract, have not been previously studied in Korea. As Korea has a mandatory universal health insurance system covering the entire population of 50 million people, which can provide all healthcare utilization related to childhood cataract and sufficient population at risk^6,7^. Therefore, in this study, we investigated the incidence of congenital and infantile (CI) cataract in Korea using the nationwide database covering the entire population.

## Results

A total of 989 CI cataract patients who underwent cataract surgery before the age of 5 years were identified among a total of 4,211,459 people born in the study period from 2003 to 2011. The number of patients by birth year and year of surgery is summarized in Table 1. The cumulative incidence of true CI (diagnosed at the age 1 or less), possible CI (diagnosed at the age 3 or less), and probable CI (diagnosed at the age 5 or less) was 1.62 (1.46–1.73), 2.38 (2.19–2.58) and 2.95 (2.66–3.23) per 10,000 births, respectively. Bilateral cataract accounted for 18.0% to 21.7% and there was no significant difference in bilateral cataract proportion between these 3 cohorts. The cohort including older patients tended to have a higher proportion of primary IOL insertion (20.1%, 39.2%, and 53.6% for true, possible, and probable CI cohorts, respectively, P < 0.01 in all comparisons). Detailed information is provided in Table 2.

**Table 1:**
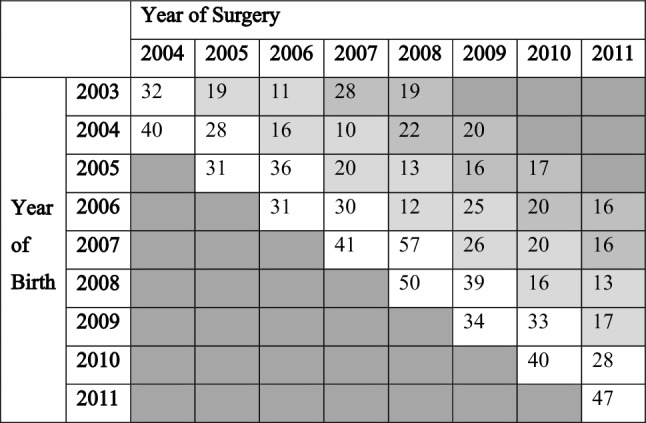
Annual number of patients with congenital and infantile cataract requiring surgery.

**Table 2:** Cumulative incidence rate and characteristics of congenital and infantile cataract according to age definition from birth to 1 year old, 3-year-old, and 5-year-old.

	True congenital and infantile cataract (0–1 year old)	Possible congenital and infantile cataract (0–3 years old)	Probable congenital and infantile cataract (0–5 years old)
Subject birth cohorts	2004–2010	2004–2008	2004–2006
Total population of cohorts	3,245,158	2,330,138	1,367,424
Female population	1,567,378	1,124,610	657,887
Male population	1,677,780	1,205,528	709,537
Total patients of cataract surgery	518	554	403
Female patients	235	251	171
Male patients	283	303	232
Total incidence rate per 10,000 birth (95% CI)	1.60 (1.46–1.73)	2.38 (2.18–2.58)	2.95 (2.66–3.23)
Female incidence rate	1.50 (1.31–1.69)	2.23 (1.96–2.51)	2.60 (2.21–2.99)
Male incidence rate	1.69 (1.49–1.88)	2.51 (2.23–2.80)	3.27 (2.85–3.69)
Laterality			
Bilateral cataract	93 (18.0%)	120 (21.7%)	81 (20.1%)
Unilateral cataract	425 (82.0%)	434 (78.3%)	322 (79.9%)
Primary IOL implantation	104 (20.1%)	217 (39.2%)	216 (53.6%)

*CI* cataract, congenital and infantile cataract, *IOL* intraocular lens.

**Figure 1:**
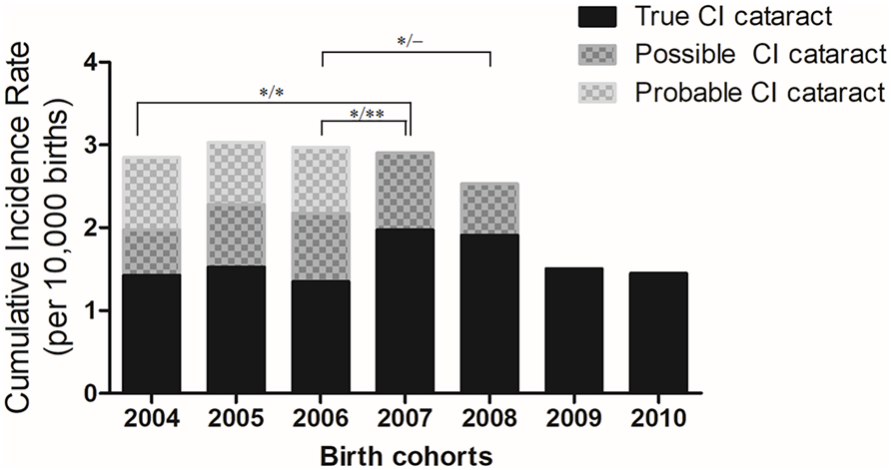
Cumulative incidence of congenital and infantile cataract by annual birth cohorts. Compared to 2004 and 2006, there was a significant increase in the incidence rates of true congenital and infantile (CI) cataracts and possible CI cataracts in 2007 (P = 0.02 for both true CI cataract and possible CI cataract compared to 2004, P = 0.04 for true CI cataract and P = 0.003 for possible CI cataract compared to 2006) Additionally, the incidence rate of true CI cataracts in 2008 showed a significant difference compared to 2006 (P = 0.04). This outcome might have been influenced by a government-led screening program launched in 2007. Yearly significant differences in incidence rates are displayed at the top of the graph in the format 'P value for true CI cataract difference/P value for possible CI cataract,' with '*' denoting P < 0.05 and '**' denoting P < 0.01.

The incidence rates for each birth year are summarized in Fig. 1 and Table 3. The incidence of true CI cataract ranged from 1.35 to 1.97 over the seven-year period from 2004 to 2010. Notably, 2007 and 2008 exhibited higher incidence rates compared to other years, and statistically significant differences were observed between 2006–2007 (P = 0.04), 2006–2008 (P = 0.04), and 2004–2007 (P = 0.02). The incidence rate of possible CI cataracts ranged from 1.97 to 2.90 over the five-year period from 2004 to 2008, with the highest rate observed in 2007. Statistically significant differences were found between 2006 and 2007 (P < 0.01). The incidence rate of probable CI cataracts ranged from 2.85 to 3.03 over the three-year period from 2004 to 2006. There were no statistically significant differences observed during these three years.

**Table 3:** Number of annual birth cohorts and cumulative incidence of congenital and infantile cataract by annual birth cohorts.

Year of birth	2004	2005	2006	2007	2008	2009	2010
Number of total births	476,958	438,707	451,759	496,822	465,892	444,849	470,171
True congenital and infantile cataract (0–1 years)							
Number of patients	68	67	61	98	89	67	68
Cumulative incidence rate^†^	1.43	1.53	1.35	1.97	1.91	1.51	1.45
Possible congenital and infantile cataract (0–3 years)							
Number of patients	94	100	98	144	118		
Cumulative incidence rate^†^	1.97	2.28	2.17	2.90	2.53		
Probable congenital and infantile cataract (0–5 years)							
Number of patients	136	133	134				
Cumulative incidence rate^†^	2.85	3.03	2.97				

^†^Per 10,000 births.

We analyzed all CI cataract patients by dividing them into two groups according to their bilaterality. Of the total 989 patients, 199 (20.1%) had bilateral cataract surgery. The mean age at the time of cataract surgery was 1.88 ± 1.60 years for the bilateral group and 1.53 ± 1.59 years for the unilateral group (P = 0.01). The age at the time of secondary IOL implantation surgery was significantly older in bilateral cataract patients (3.77 ± 2.46 vs 2.51 ± 1.22 years, P < 0.0001). In the unilateral cataract group, the proportion of patients who underwent strabismus surgery within 8 years after cataract surgery was statistically significantly higher (31.4% vs. 21.6%, P = 0.01). The proportion of patients who underwent glaucoma surgery was 11 (5.5%) in the bilateral cataract group and 21 (2.7%) in the unilateral cataract group, but it did not reach statistical significance (P = 0.07) (Table 4).

**Table 4:** Baseline characteristics, additional diagnosis, and additional surgical treatment after cataract surgery by laterality.

	Bilateral cataract	Unilateral cataract	P value
Total patients	199/989 (20.1%)	790/989 (79.9%)	
Age at cataract surgery	1.88 ± 1.60	1.53 ± 1.59	0.01
Sex			0.63
Female	83 (41.7%)	347 (43.9%)	
Male	116 (58.3%)	443 (56.1%)	
Primary IOL	117/199 (58.8%)	322/790 (40.8%)	
Age at primary IOL	2.82 ± 1.55	2.75 ± 1.52	0.68
Female	50 (42.7%)	141 (43.8%)	0.91
Secondary IOL	30/199 (15.1%)	264/790 (33.4%)	
Age at secondary IOL	3.77 ± 2.46	2.51 ± 1.22	< 0.0001
Female	12 (40.0%)	127 (48.1%)	0.44
Aphakia	52/199 (26.1%)	204/790 (25.8%)	
Female	21 (40.4%)	79 (38.7%)	0.87
Additional diagnosis after cataract surgery			
Strabismus	161 (80.9%)	664 (84.1%)	0.29
Glaucoma	142 (71.4%)	550 (69.6%)	0.67
Additional surgery after cataract surgery			
Strabismus surgery	43 (21.6%)	248 (31.4%)	0.01
Glaucoma surgery	11 (5.5%)	21 (2.7%)	0.07

Among the total of 989 CI cataract patients, 439 (44.4%) underwent primary IOL implantation, 294 (29.7%) underwent secondary IOL implantation, and the remaining 256 individuals (25.9%), who did not undergo IOL implantation within 8 years after cataract surgery, were categorized as the aphakia group. The mean age at the time of cataract surgery was 2.66 ± 1.53 years in the primary IOL group, 0.63 ± 0.91 years in the secondary IOL group, and 0.89 ± 1.20 years in the aphakia group, and it showed statistically significant differences in all three groups (P < 0.0001). The mean age of the patients who underwent secondary IOL implantation was 2.64 ± 1.44 years. The secondary IOL group had a significantly higher proportion of unilateral cataracts compared to the other two groups (P < 0.0001). The secondary IOL group exhibited significantly higher rates of unilateral cataract, glaucoma diagnosis, and strabismus surgery compared to the other two groups (P < 0.0001 in all comparisons). The diagnosis rate for strabismus differed significantly among all three groups, with the highest rate in the secondary group, followed by the primary group, and the lowest rate in the aphakia group (P < 0.0001). Furthermore, the primary IOL group exhibited a significantly lower rate of glaucoma surgery compared to the other two groups (P < 0.01) (Table 5).

**Table 5:** Baseline characteristics, additional diagnosis, and additional surgical treatment after cataract surgery by the type of intraocular lens implantation.

	Primary IOL	Secondary IOL	Aphakia*	P value
Total patients (%)	439 (44.4%)	294 (29.7%)	256 (25.9%)	
Age at cataract surgery	2.66 ± 1.53	0.63 ± 0.91	0.89 ± 1.20	< 0.0001^†^
Age at 2nd IOL	NA	2.64 ± 1.44	NA	
Sex				0.16
Female	191 (43.5%)	139 (47.3%)	100 (39.1%)	
Male	248 (56.5%)	155 (52.7%)	156 (60.9%)	
Laterality				< 0.0001^‡^
Bilateral	117 (26.7%)	30 (10.2%)	52 (20.3%)	
Unilateral	322 (73.3%)	264 (89.8%)	204 (79.7%)	
Additional diagnosis after cataract surgery				
Strabismus	361 (82.2%)	283 (96.3%)	181 (70.7%)	< 0.0001^§^
Glaucoma	281 (64.0%)	252 (85.7%)	159 (62.1%)	< 0.0001^∥^
Additional surgery after cataract surgery				
Strabismus surgery	120 (27.3%)	122 (41.5%)	49 (19.1%)	< 0.0001^¶^
Glaucoma surgery	5 (1.1%)	15 (5.1%)	12 (4.6%)	< 0.01**

*Patients who did not undergo IOL implantation within 8 years after cataract surgery.

^†^P < 0.0001 between primary and secondary groups, P < 0.0001 between primary and aphakia groups, and P < 0.01 between secondary and aphakia groups.

^‡^P < 0.0001 between primary and secondary groups, P < 0.01 between secondary and aphakia groups, and P = 0.20 between primary and aphakia groups.

^§^P < 0.0001 between primary and secondary groups, P < 0.0001 between secondary and aphakia groups, and P < 0.01 between primary and aphakia groups.

^∥^P < 0.0001 between primary and secondary groups, P < 0.0001 between secondary and aphakia groups, and P = 1.0 between primary and aphakia groups.

^¶^P < 0.001 between primary and secondary groups, P < 0.0001 between secondary and aphakia groups, and P = 0.051 between primary and aphakia groups.

**P < 0.01 between primary and secondary groups, P = 1.00 between secondary and aphakia groups, and P = 0.02 between primary and aphakia groups.

## Discussion

We presented the incidence rate of congenital and infantile cataracts using the NHIS database covering the entire healthcare utilization from the Korean population of 50 million people. We defined three types of cumulative incidence rates for true CI cataract, possible CI cataract, and probable CI cataract with the age criteria of 1, 3 and 5 years old, respectively. The corresponding cumulative incidence rates per 10,000 births in each group were 1.60, 2.38, and 2.95, respectively. Rahi et al.^8^ reported the cumulative incidence rates of infantile cataract per 10,000 persons as 2.49, 3.18, and 3.46, respectively, based on the diagnostic age of 1, 5, and 15 years of age. Holmes et al.^9^ analyzed the infantile cataract (before 1 year of age) and the possible infantile cataract (before the age of 8) and reported the cumulative incidence rate per 10,000 births as 3.0 and 4.5, respectively. In these studies, about only one-third of patients were diagnosed after 1 year of age, which is quite lower than the present results; about 45% of patients were diagnosed after 1 year of age. The discrepancy suggests that incidence rates estimated only from infants (before 1 year of age) might underestimate the overall social burden of CI cataracts.

Primary and secondary IOL implantation were performed in 44% and 30% of all patients in the present study. In the remaining 26% of patients, IOL implantation was not performed within 8 years after cataract surgery. When IOLs should be implemented in CI cataract has been a long-standing debate concerning visual prognosis, binocular function, ocular alignment, complications, and medical costs^10–16^. Lambert et al. did not recommend IOL implantation in infants 6 months of age or younger because of the high incidence of postoperative visual axis opacities^17^. Zhao et al. reported that age at surgery and laterality of cataract were two important factors when judging the optimal timing of IOL implantation. It was also recommended to delay the IOL implantation to reduce postoperative complications in a bilateral cataract^18^. The present results are similar to the recommendations of these literatures; children who underwent primary IOL implantation were significantly older than the others, and secondary IOL implantation was performed later in the bilateral cataract cases compared to the unilateral cataract cases.

After cataract surgery, strabismus was diagnosed in 83% of CI cataract patients, and strabismus surgery was performed in 29% of the total cohort. Previous studies have reported a range of strabismus co-occurrence rates in children with cataracts, ranging from 20.5 to 86%^19^. Our findings suggest a slightly higher strabismus diagnosis rate but generally align with existing research. Glaucoma diagnosis and surgery occurred in 70% and 3% of all CI cataract patients, which is much high in diagnosis compared to previous studies and a meta-analysis that reported a 17% occurrence rate^20^. Given the potential for overestimating incidence rates when relying solely on diagnostic codes in claims data, surgical occurrence rates using surgical codes are considered more reliable indicators, particularly for our subgroup analyses. Previous research has identified younger age, unilateral cases, and low postoperative visual acuity as risk factors for postoperative strabismus^19,21^, and younger age, primary IOL implantation, additional intraocular surgery, and bilateral cases as risk factors for postoperative glaucoma^20,22^. Our subgroup analyses largely align with prior findings, although there are some differences. The variability in results across studies on these risk factors suggests the need for additional research in this area, especially since this study design did not include multivariate analysis for assessing these risk factors.

It is particularly intriguing that in our study, true and possible congenital cataracts (CI) increased in numbers in 2007 and 2008. This is of significant interest because this coincides with the initiation of the National Health Screening Program for Infants and Children (NHSPIC) in Korea in 2007^23^, which included vision screening. The introduction of such a national screening program can potentially lead to an increase in diagnosis rates and the number of surgical cases. A similar phenomenon has been reported in middle ear and cochlear implant surgeries in pediatric patients^24^. However, it's noteworthy that the incidence of true CI cataracts in 2009 and 2010 decreased again and did not show significant differences from the preceding years. In a previous study analyzing developmental disorders within NHSPIC, an increasing trend in false negative proportions in the screening results (from 2.7% in 2008 to 30.2% in 2015) was reported, which could also be a contributing factor to our findings. The specific cause for the increasing false negative proportion has not been documented. Given the rapid increase in the screening rate, and the fact that primary care pediatricians must assess all aspects of an infant's development within a short time, and vision screening for children under 2 years of age relies solely on parent-completed questionnaires and penlight examinations by general pediatricians rather than ophthalmologists^25^, there is a possibility that screening fatigue may have had an impact. Taking into consideration the NHSPIC's screening rate increase from 35.1% in 2008^26^ to 83% in 2020(KOSIS, https://kosis.kr, accessed on April 1, 2022), it is anticipated that the year-to-year changes in diagnosis rates for CI cataracts may yield intriguing findings in subsequent research when additional follow-up data becomes available.

The present study has several limitations. As we analyzed the claims database which lacked comprehensive eye examinations, we defined the CI cataract patients by identifying all patients who underwent cataract surgery before the age of 5 years and by excluding other conditions requiring cataract surgery including trauma, congenital glaucoma, etc. When confined to the claims in only patients with a diagnosis of congenital cataract (Q12.0) or infantile cataract(H26,0), only about 50% of the patients were included because of the ‘diagnoses downcoding’ that often occurs in the real world. Therefore, we assumed that this could lead to an underestimation of CI cataract prevalence. Fortunately, a previous large cohort study conducted with a comprehensive chart review revealed that CI cataract was the most common in children up to 3 years of age (over 99% of all types of cataracts). Even up to age 9, CI cataract occupied the largest portion of incidence^27^. Therefore, the impact of our method is not considered to be significant. Furthermore, for similar reason, we had another limitation in documenting the frequently co-occurring various factors in CI cataract patients, such as genetic comorbidities, metabolic conditions, infectious diseases, and other ocular anomalies. Finally, because we identified only patients requiring surgery, the prevalence may be underestimated compared to the entire CI cataract patient population.

Notwithstanding these limitations, the present study has several strengths. To the best of our knowledge, the analyzed birth cohort which included 4,211,459 births is the largest study of childhood cataract so far, and the present study is the first epidemiologic study regarding childhood cataract in Korea. In addition, we also analyzed at least 8 years after cataract surgery in each patient using the nationwide claims database which includes all healthcare utilization without omission. Therefore, we could conduct detailed, comprehensive analyses not only for the subgroups according to IOL status and laterality but also for additional diagnoses and following surgeries among the patients with CI cataract. As the Korean government has provided a universal, compulsive medical insurance system, the NHIS, to the entire Korean population, Korea is one of the countries with the highest access to health care and provides the highest level of medical services including ophthalmology^28^. In addition, as Korea is one of the most populous countries, it might be best for investigating the incidence rate of diseases with low incidence, such as CI cataract.

In conclusion, the incidence rates of CI cataract in Korea appear to be comparable to previous studies in other regions. The early screening program for infants may reduce such delayed diagnosis and increase the proportion of patients undergoing surgery at a critical time for visual development. Further study is warranted on whether the government-led screening program for CI cataract can detect CI cataract early and improve the visual prognosis of CI cataract patients.

## Methods

We conducted the present study using the national claims database of the National Health Insurance Service (NHIS) of Korea. The NHIS scheme started in Korea in 1977 and achieved universal coverage of the entire Korean population by 1989. After being integrated into the only insurer in 2000, the NHIS has become mandatory for all Korean residents and healthcare providers. Therefore, the NHIS database contains records for about the entire 50 million Korean residents including demographic information, all medical claims for diagnoses, procedures, prescription records, and direct medical costs. The NHIS database codes diagnoses according to the Korean version of the International Classification of Diseases, 10th edition (ICD-10), the Korean Standard Classification of Diseases (KCD). The NHIS database is provided to Researchers who have been approved for the research protocol by the official review committee, excluding personally identifiable information. Additional information on NHIS and its database has been reported in detail elsewhere^6,7,29–31^. This study was approved, and informed consent was waived by the institutional review board of the Seoul National Bundang Hospital (IRB no.: X-2203-744-901) because NHIS provided de-indentificated data. This study also adhered to the tenets of the Declaration of Helsinki.

We analyzed the NHIS database set, which contained all claims from 2002 to 2019 for patients who underwent any cataract surgery [surgical code: S5119 (phacoemulsification) or S5111(extracapsular or intracapsular lens extraction)] between 2004 and 2011. Among these patients, we included those born between 2003 and 2011 and excluded those with traumatic cataract (H26.1), complicated cataract (H26.2, H26.3, H28.0), congenital glaucoma (Q15), and any history of eye-related trauma identified by diagnostic code of S05 and H40.3. Therefore, we could analyze all medical utilization of included patients at least 8 years after cataract surgery. As the NHIS did not allow to access detailed information regarding the exact birth date of each identified patient, we could access only the year of the birth for each patient. Therefore, we tabulated the number of identified patients according to their birth year and the year of the surgery (Table 1). We defined three CI cataracts within the scope of our data: ‘true’ CI cataract, ‘possible’ CI cataract, and ‘probable’ CI cataract. True CI cataract is defined as cataract surgery between 0 and 1 years of age, possible CI cataract as cataract surgery between 0 and 3 years of age, and probable CI cataract as cataract surgery between 0 and 5 years of age. For example, in the 2004 birth cohort, true CI cataract patients included patients operated in 2004 and 2005. In extreme cases, this definition can include up to 23 months of age of operation. But considering the median birth and surgery date of the birth cohort and time from diagnosis to surgery, it was considered the most consistent with the traditional definition in literature^32^. Possible and probable CI cataracts were also defined as patients who underwent surgery between 2004 and 2007 and between 2004 and 2009 in the 2004 birth cohort, respectively, and these can be considered as a number including delayed diagnosis. Since our database included patients who underwent any cataract surgery between 2004 and 2011, we were able to calculate the incidence of true CI cataract from the 2004 birth cohort to the 2010 birth cohort, covering a total of seven years of birth cohorts. Similarly, the possible cataract could be calculated for 5-year birth cohorts (from the 2004 birth cohort to the 2008 birth cohort), and the probable CI cataract could be calculated for 3-year birth cohorts (from the 2004 birth cohort to the 2006 birth cohort). Then we calculated the cumulative incidence rate for each definition using publicly available birth data from the Korea Statistical Information Service (KOSIS, https://kosis.kr, accessed on November 1, 2021). We also calculated a 95% confidence interval for each cumulative incidence based on the Poisson distribution. Laterality and IOL implantation type were also analyzed using surgical codes of IOL implantation [S5116 (primary IOL implantation), S5117(secondary IOL implantation)]. In addition, newly generated diagnoses and additional surgeries during 8 years after cataract surgery were identified to analyze ocular complications.; glaucoma (H40) and strabismus (H50, H05.9) were identified by using the diagnostic codes and surgeries including strabismus surgery (S5173 to S5178), and glaucoma surgery (S5040 to S5049) were assessed by the surgical codes.

For statistical analyses, distribution normality was assessed using the Kolmogorov–Smirnov test. Continuous data were presented as mean ± (standard deviation), while categorical variables were presented as frequency (%). Comparisons of continuous variables between two groups were performed using the T-test, while comparisons among three groups were conducted using ANOVA. Categorical variables were assessed using Fisher's exact test. Statistical analyses were carried out using R language version 4.1.2 (R Foundation for Statistical Computing, Vienna, Austria) for the other analyses. P-values < 0.05 were considered statistically significant.

## Data Availability

The datasets generated and analyzed during the current study are available in the National Health Insurance Sharing Service (NHISS) of Korea after approval of data acquisition, https://nhiss.nhis.or.kr/.
